# Bacterial infection profiles in lung cancer patients with febrile neutropenia

**DOI:** 10.1186/1471-2334-11-183

**Published:** 2011-06-27

**Authors:** Jean-Philippe Lanoix, Emilie Pluquet, Francois Xavier Lescure, Houcine Bentayeb, Emmanuelle Lecuyer, Marie Boutemy, Patrick Dumont, Vincent Jounieaux, Jean Luc Schmit, Charles Dayen, Youcef Douadi

**Affiliations:** 1 Department of Infectious Diseases, Amiens University Medical Center, Place Victor Pauchet, F-80000 Amiens, France; 2 Department of Pneumology, Tourcoing Medical Center, 135 rue Pres Coty, F-59200 Tourcoing, France; 3 Department of Infectious Diseases, Tenon Hospital, 4 rue de Chine, F-75020 Paris, France; 4 Department of Pneumology, Saint-Quentin Medical Center, 1 avenue Michel de l'hospital, F-02100 Saint-Quentin, France; 5 Department of Pneumology, Chauny Medical Center, 94 rue des Anciens combattants, F-02300 Chauny, France; 6 Department of Pneumology, Amiens University Medical Center, avenue Laennec, F-80000 Amiens, France

## Abstract

**Background:**

The chemotherapy used to treat lung cancer causes febrile neutropenia in 10 to 40% of patients. Although most episodes are of undetermined origin, an infectious etiology can be suspected in 30% of cases. In view of the scarcity of data on lung cancer patients with febrile neutropenia, we performed a retrospective study of the microbiological characteristics of cases recorded in three medical centers in the Picardy region of northern France.

**Methods:**

We analyzed the medical records of lung cancer patients with neutropenia (neutrophil count < 500/mm^3^) and fever (temperature > 38.3°C).

**Results:**

The study included 87 lung cancer patients with febrile neutropenia (mean age: 64.2). Two thirds of the patients had metastases and half had poor performance status. Thirty-three of the 87 cases were microbiologically documented. Gram-negative bacteria (mainly enterobacteriaceae from the urinary and digestive tracts) were identified in 59% of these cases. *Staphylococcus *species (mainly *S. aureus*) accounted for a high proportion of the identified Gram-positive bacteria. Bacteremia accounted for 60% of the microbiologically documented cases of fever. 23% of the blood cultures were positive. 14% of the infections were probably hospital-acquired and 14% were caused by multidrug-resistant strains. The overall mortality rate at day 30 was 33% and the infection-related mortality rate was 16.1%. Treatment with antibiotics was successful in 82.8% of cases. In a multivariate analysis, predictive factors for treatment failure were age >60 and thrombocytopenia < 20000/mm^3^.

**Conclusion:**

Gram-negative species were the most frequently identified bacteria in lung cancer patients with febrile neutropenia. Despite the success of antibiotic treatment and a low-risk neutropenic patient group, mortality is high in this particular population.

## Background

Lung cancer is the most frequent and severe cancer in France. In 2000, the incidence was 52.2 per 100,000 inhabitants per year [1]. Although lung cancer mainly affects male, middle-aged smokers, the recent increase in the number of female smokers is changing these data [2]. The choice of treatment depends on the lung cancer's histological characteristics. When chemotherapy is used, neutropenia (i.e. a neutrophil count < 500/mm^3^) of variable severity and duration occurs in 10 to 40% of patients (depending on the type of chemotherapy) [3,4]. In solid tumors, neutropenia usually lasts for less than 7 days. However, the condition can be complicated by fever (a body temperature of over 38.3°C once an hour or over 38°C twice an hour) in 8 to 15% of cases and is thus referred to as febrile neutropenia [5,6]. Microbiological evidence is found in only 30% of patients with neutropenic fever. Indeed, approximately 60% of patients with febrile neutropenia have neither clinical signs nor microbiological evidence of infection [7].

Prior to the 1980s, neutropenic fever was due to Gram-negative bacteria (e.g. *Escherichia coli, Klebsiella *sp, *Pseudomonas aeruginosa, etc*.) in 70% of cases [8,9]. Since then, the proportion of Gram-positive bacteria has risen, with *Staphylococcus epidermidis *being found more frequently than *Staphylococcus aureus *[10].

Lung cancer patients are characterized by the frequent presence of *Pseudomonas aeruginosa *in chronic obstructive pulmonary disease, together with age-related comorbidities, aggressive tumors and rapid clinical degradation.

Markman and Abeloff (1983) studied the infectious complications of chemotherapy (with cyclophosphamide, doxorubicin and etoposide) in 71 patients with small-cell carcinoma [11]. Although 90% of cases were complicated by febrile neutropenia, an etiology was determined in only 20% of the latter. The following bacteria were found: *Escherichia coli *(n = 3), *Streptococcus viridans *(n = 2), *Pseudomonas aeruginosa *(n = 2), *Bacillus *sp. (n = 2), *Staphylococcus aureus *(n = 1), *Candida *sp. (n = 1), *Enterococcus *sp. (n = 1) and one anaerobic Gram-negative bacillus. None of these cases of febrile neutropenia resulted in death.

Two other studies in lung cancer patients examined the outcomes of antibiotic therapy rather than the microbiological characteristics of the condition [12,13]. No statistically significant differences were found.

Here, we performed a retrospective, descriptive, multicenter study of the microbiological characteristics of febrile neutropenia in lung cancer patients hospitalized in three medical centers in the Picardy region of northern France. The study's primary objective was to determine risk factors for antibiotic treatment failure.

## Methods

We included adult lung cancer patients (i.e. aged 18 or over) having undergone chemotherapy (of whatever type) between January 2000 and July 2006 in three medical centers in the Picardy region. Patients had to have displayed concomitant neutropenia (i.e. a neutrophil count < 500/mm^3^) and fever (a body temperature > 38.3°C once an hour or over 38°C twice an hour). Access to the patients' medical records was provided by the medical centers' health informatics departments.

We excluded patients who had already experienced an episode of febrile neutropenia prior to study entry, patients with cancers other than lung cancer, cases of fever without neutropenia, cases of neutropenia without fever and cases of fever with a clearly non-infectious etiology.

Data was recorded in a pre-determined questionnaire. Febrile episodes were classified according to Hughes' criteria as being microbiologically documented, clinically documented or of undetermined origin. The success of antibiotic therapy was defined according to Kern's criteria: no fever for 3 successive days, the absence of clinical signs or the eradication of an identified pathogen. The death of a patient meeting these criteria was not considered to be a treatment failure [14]. The last criterion for success was the lack of recurrence within 7 days of the end of antibiotic therapy. We calculated the "first-line antibiotic treatment failure rate" (i.e. failure of the first-line antibiotic prescribed) and the "overall antibiotic treatment success rate" (i.e. a successful outcome when one or more antibiotics had been prescribed).

We also recorded the presence of the following severity criteria on admission: systolic blood pressure < 90 mmHg, blood oxygen partial pressure < 60 mmHg, disseminated intravascular coagulation, confusion, cardiac failure, blood transfusion for hemorrhage, kidney failure and admission to the intensive care unit [15].

Data were gathered in compliance with the principles of the Declaration of Helsinki and then anonymized. No patient consent was needed because of the confidential nature of this study, which was approved by the independent ethics committee at Saint-Quentin Medical Center.

Statistical analysis was performed with Excel^® ^2000 (Microsoft EMEA, Courtaboeuf, France) and SPSS (SPSS Inc., Chicago, IL) software. Quantitative variables were expressed as the mean ± standard deviation and qualitative variables were expressed as the frequency and percentage. Univariate, comparative analyses were performed by using Fisher's exact test (for qualitative variables) and the Mann-Whitney test (for quantitative variables). The threshold for statistical significance was set to p < 0.05. Multivariate analyses were performed by using logistic regression.

## Results

**Figure 1:**
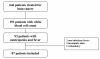
**Flow charts**.

Over the study period, 646 patients were treated for lung cancer. Ninety-three of these patients (14%) fulfilled the inclusion criteria (i.e. hospitalized for febrile neutropenia) and 6 were excluded (2 cases of fever with a non-infectious etiology, 3 patients with incomplete data and 1 patient enrolled twice). Details are shown in Figure 1 and 87 episodes were analyzed. The study population (mean age: 64.2 ± 9.6) was generally comprised of male (male/female ratio: 8:1) smokers (95%, n = 83). Chronic obstructive pulmonary disease was recorded in 53.5% of cases (n = 46) (for details, see Table 1). Half of these patients had poor performance status and 14% had been hospitalized prior to the episode of febrile neutropenia.

**Table 1:** Characteristics of patients

	Number *(%) *or means *± SD*
Sex (M/F)	78/9
Age (year)	64.2 *± 9.6*
Score WHO ≥ 2	54/86 *(62.8)*
Allergy (penicillin)	2/87 *(2.3)*
Comorbidity	53/87 *(61)*
- diabetes	17 *(19.5)*
- therapy by corticosteroids	24 *(27.6)*
- immunosuppression	2 *(2.3)*
- undernutrition	5 *(5.7)*
- other cancer	10 *(11.5)*
- other comorbidity	10 *(11.5)*
No of comorbidities	1.3 *± 0.5*
No. of comorbidities ≥ 2	11/87 *(12.6)*
Smoking	83/87 *(95.4)*
No of packets × No of year of smoking	46.5 *± 19.5*
History of pulmonary diseases	58/86 *(67.4)*
- asbestos	9 *(10.5)*
- asthma	1 *(1.2)*
- COPD	46 *(53.5)*
- restrictive syndrome	7 *(8.1)*
- bronchiectasis	2 *(2.3)*
- oxygenotherapy	2 *(2.3)*
- pulmonary tuberculosis	2 *(2.3)*
State COPD	39/86
- Normal	2 *(2.3)*
- FEV1 > 80% of theory	10 *(11.6)*
- FEV1 between 50 and 80% theory	17 *(21)*
- FEV1 between 30 and 50% theory	5 *(7)*
- FEV1 < 30% of theory	3 *(3.5)*

COPD = Chronic Obstructive Pulmonary Disease, FEV1 = Forced Expiratory Volume in first second, No = number.

Almost two thirds of the patients had metastatic cancer when they were diagnosed with febrile neutropenia (62% in all, including brain metastases in 14% and metastases at more than two sites in 20%). Thirty-one percent of the non-metastatic patients were non-operable and 28.7% (n = 25) of patients had also been treated with radiotherapy (data not shown). Eighty patients (92%) had an implantable venous access system and 15 patients (17%) had undergone two or more cycles of chemotherapy.

Thirteen percent of the patients had febrile neutropenia (87 out of 646). Sixteen events were excluded because they did not correspond to the patient's first ever occurrence of febrile neutropenia. The mean duration of neutropenia was 4.4 ± 2.4 days (range: 1-11) and 10% of the episodes lasted more than 8 days.

The fever was classified as being of undetermined origin in 46% of cases (n = 40), clinically documented in 45% of cases (n = 39) (including 14 (16%) with no microbiology documentation) and microbiologically documented in 38% of cases (n = 33). Three quarters of the microbiologically documented cases (n = 25 out of 33) were also clinically documented. The clinically documented infections were classified as follows: 21 lower respiratory tract infections (53%), 9 digestive infections (23%), 3 catheter infections (8%), 3 skin infections (8%), 2 urinary tract infections and one upper respiratory tract infection.

Of the 33 patients with identified pathogens, 7 (21.2%) had two different bacterial infections and one had 3. Most of the identified germs were Gram-negative bacteria (n = 24; 59%) and most of the latter were Enterobacteriaceae (especially *Escherichia coli) *(n = 11 out of 16). Most of the Gram-positive bacteria (n = 17) were *Staphylococcus *sp. (above all *S. aureus*; n = 6 out of 9). Details are shown in Table 2. Neither Gram-positive cocci nor Gram-negative bacilli were associated with mortality (p = 0.43 and p = 1, respectively). However, infection with Gram-negative bacilli was significantly associated with first-line antibiotic treatment failure in cases of microbiologically documented fever (9 out of 21 successes vs. 10 out of 12 failures; p = 0.02).

**Table 2:** Microbiological characteristics of bacteria strains

Classification n *(%) *	Details n
**Gram-positive bacteria**: 17 *(41%)*	*Staphylococcus *sp: 9
	*S. epidermidis*: 3 (methi-S: 1 - methi-R: 2)
- Gram positive Cocci: 16	*S. aureus*: 6 (methi-S: 4 - methi-R: 2)
	*Streptococcus *sp: 4
	*- Streptococcus A *: 2
	*- Streptococcus D bovis *: 1
	*- Lactococcus lactis cremoris *: 1
	*S. pneumoniae*: 2
	*Enterococcus *sp: 1
	
- Gram positive Bacilli: 1	*Turicella otitidis*: 1

**Gram-negative bacteria**: 24 *(59%)*	*H. influenzae *and *parainfluenzae*: 3
	Enterobacteriaceae: 16
- Gram negative Bacilli: 23	*- E. coli*: 11
	*- Proteus mirabilis*: 1
	*- Klebsiella oxytoca*: 2
	*- Salmonella enterica*: 1
	*- Citrobacter koseri*: 1
	*Pseudomonas aeruginosa*: 2
	*Sphingomonas paucimobilis*: 1
	*Acinetobacter baumannii*: 1
	
- Gram negative Cocci: 1	*Moraxella catarrhalis*: 1

methi-S = methicillin sensitive; methi-R = methicillin resistant; sp = species

Gram-negative bacilli predominated in both monomicrobial and polymicrobial infections. There were 13 Gram-negative monomicrobial infections and 11 Gram-positive monomicrobial infections. One diagnosis was made on the basis of serum antibodies against an intracellular bacterium, *Chlamydia pneumoniae*. In polymicrobial infections, combinations of several different Gram-negative bacteria were found in 4 cases. Combinations of several different Gram-positive species and combinations of Gram-negative and Gram-positive species were found in 2 cases each. However, we decided not to include four other doubtful combinations in which one of the bacteria was non-pathogenic and was not isolated from the same body site as the identified pathogenic species.

Multidrug-resistant bacteria (i.e. those resistant to two or more antibiotic families, including beta-lactams) were identified in 6 of the 41 infections (14.6%). Six of the 33 patients (18%) were infected with multidrug resistant bacteria. All *S. pneumoniae *were penicillin non-susceptible (n = 2) and almost half of all *Staphylococcus sp*. were methicillin-resistant (n = 4 out of 9). One of the two *Pseudomonas aeruginosa *isolates was ceftazidime-resistant and the only *Sphingomonas paucimobilis *isolate was found to be multidrug-resistant. There were no vancomycin-resistant *Enterococcus *isolates and no gentamycin-intermediate-susceptible *S. aureus *isolates. According to the antibiotic susceptibility profile, 24% of patients (n = 8) would have needed glycopeptides or cephaloridine.

The isolates were mainly urinary and digestive tract germs (46%, n = 19), followed by cutaneous (22%, n = 9) and respiratory (20%, n = 8) germs (for details, see Table 3). Blood cultures were the most frequently positive samples (23%; 20 positive blood cultures out of 81 samples) and yielded 24 of the 45 germs (including 9 *E. coli*, 4 *S. aureus *and 4 *Streptococcus *sp isolates), since 4 patients had polymicrobial bacteremia. The origin of the bacteremia could be identified in half the cases, since 13 of the 20 cases of bacteremia were associated with clinical signs. The other samples collected for bacteriological testing were sputum (n = 9), urine (n = 3), stools (n = 4), skin (n = 5) and others (n = 5). A positive blood culture was not a risk factor for mortality (p = 0.8).

**Table 3:** Repartition of germ according to their tropism.

Uro-digestives germs: 19 strains	*Enterococcus *spp: 1 Enterobacteriaceae: 16 *Acinetobacter baumannii *: 1 *Streptococcus bovis *: 1
Cutaneous germs: 9 strains	*Coagulase Negative Staphylococcus*: 3 *Staphylococcus aureus*: 6
Respiratory germs: 8 strains	*S. pneumoniae*: 2 *Haemophilus influenzae *: 3 *Pseudomonas aeruginosa*: 2 *Moraxella catarrhalis *: 1
Other: 5 strains	Other *Streptococcus*: 3 *Sphingomonas paucimobilis *: 1 *Turicella otitidis *: 1

All three centers applied the same antibiotic treatment guidelines. The first-line treatment was either amoxicillin/clavulanic acid or a combination of cephalosporin and ciprofloxacin. The concordance between the guidelines and actual prescriptions was 65%; the discordances consisted of adjunction of an aminoglycoside in 41% of cases and use of a glycopeptide in 6% of cases. The outcome was favorable in most cases; the overall antibiotic therapy success rate was 82.8%. The first-line treatment was effective in 72.4% of cases (63 out of 87) but fell to 63.6% (n = 21 out of 33) in microbiologically documented episodes. Furthermore, the outcome of first-line treatment was in line with the *in vitro *susceptibility results in 84.8% of cases (28 out of 33). The median time interval between treatment initiation and apyrexia was 2 days (range: 1-12).

The mortality rate was 15% at day 7 and 33% at day 30, whereas the infection-related mortality rate was 16.1%. Ten of the 14 deceased patients (71%) had at least one severity criterion on admission.

In a univariate analysis, the predictive factors for first-line antibiotic treatment failure were as follows: age>60, WHO score >2, thrombocytopenia below 20,000/mm^3^, undetermined origin, mismatch between microbiological results and antibiotic treatment and, lastly, hospital-acquired infections. The latter three factors did not have a significant impact on the overall success of antibiotic treatment (Table 4).

**Table 4:** Predictive factors of first-line antibiotic treatment failure.

	Failure n = 24 n *(%)*	Success n = 63 n *(%)*	p value
Mean age (years)	69.2	62.33	**<0.001**
Age >60 years	20 *(83.3)*	32 *(50.8)*	**0.007**
WHO Score ≥2	20 *(83.3)*	34 *(54)*	**0.024**
No. of comorbidities>2	4 *(16.7)*	7 *(11.1)*	0.487
Diabetes	7 *(29.2)*	10 *(15.9)*	0.225
Corticosteroid treatment	9 *(37.5)*	15 *(23.8)*	0.283
Pulmonary antecedents	15 *(62.5)*	43 *(68.3)*	0.800
COPD	14 *(58.3)*	32 *(50.8)*	0.470
Severe COPD (FEV1<30% theory)	1 *(4.2)*	2 *(3.2)*	1
Small cell cancer	6 *(25)*	17 *(27)*	1
More than 1 chemotherapy	4 *(16.7)*	10 *(15.9)*	1
Metastases	18 *(75)*	36 *(57.1)*	0.145
Radiotherapy associated	6 *(25)*	19 *(30.2)*	0.793
G-CSF preventive	4 *(16.7)*	13 *(20.6)*	0.771
G-CSF curative	12 *(50)*	19 *(30.2)*	0.132
Already hospitalized patient	7 *(29.2)*	5 *(7.9)*	**0.017**
Altered general status	17 *(70.8)*	30 *(47.6)*	0.059
Dehydration	14 *(58.3)*	20 *(31.7)*	**0.029**
Gravity signs at admission	15 *(62.5)*	29 *(46)*	0.231
Delayed treatment	6 *(25)*	12 *(19)*	0.562
Neutropenia > 8 days	3 *(12.5)*	6 *(9.5)*	0.702
Neutrophils count <100/mm3	7 *(29.2)*	13 *(20.6)*	0.354
Haemoglobin <8 g/dl	9 *(37.5)*	32 *(50.8)*	0.465
Platelets < 20000/mm3	10 *(41.7)*	8 *(12.7)*	**0.006**
Decreasing of CRP	8 *(33.3)*	32 *(50.8)*	**0.002**
Undetermined origin of fever	6 *(25)*	34 *(54)*	**0.018**
Clinically documented fever (without microbiological documentation)	6 *(25)*	8 *(12.7)*	0.196
Microbiologically documented fever	12 *(50)*	21 *(33.3)*	0.216
Respiratory signs	6 *(25)*	15 *(23.8)*	0.203
Bacteraemia	9 *(37.5)*	11 *(17.5)*	0.277
Gram negative bacilli	10 *(41.7)*	9 *(14.2)*	**0.03**
Multidrug resistant bacteria in patient with at least one strain documented	4 *(33.3)*	2 *(9.5)*	0.159
Antibiotic treatment in last 6 month	6 *(25)*	16 *(25.8)*	1
Out patient first antibiotic treatment	4 *(16.7)*	10 *(15.9)*	1
Appropriateness between microbiology and antibiotherapy in patient with at least one strain documented	7 *(58.3)*	21 *(100)*	**0.003**

COPD = Chronic Obstructive Pulmonary Disease, CRP = C-reactive protein, FEV1 = Forced Expiratory Volume in first second, G-CSF = Granulocyte colony-stimulating factor, No = number.

A multivariate analysis revealed that three factors were predictive of first-line antibiotic treatment failure: age>60 (p = 0.011), thrombocytopenia <20,000/mm^3 ^(p < 0.01) and WHO score >2 (p = 0.012). The first two factors were also correlated with the overall failure of antibiotic treatment.

## Discussion

As expected, our population was typical of hospitalized lung cancer patients and primarily comprised elderly male smokers in poor general health (WHO score >2). The recent increase in the number of female smokers in France did not appear to have impacted on the present study. Likewise, the patients' prognoses were very poor, with a 33% overall mortality rate (half of which was due to febrile neutropenia) on day 30. In comparison with other studies, the mortality rate was high but may be explained by the fact that our study population was particularly severely affected: 60% had metastases and 31% were non-operable [14,16].

Surprisingly, most of the identified bacteria were germs of the urinary and digestive tracts (rather than respiratory germs) and were predominantly detected by blood culture. In contrast, the clinically documented infections were mostly due to respiratory germs (53%, vs. 23% for digestive tract infections). These findings suggest that respiratory germs rarely cause bacteremia. Fifteen percent of the infections were multidrug-resistant and only 14% were hospital-acquired infections; these are low values for a population which is frequently hospitalized. However, multidrug-resistance and hospital-acquired infection did not appear to have a significant impact on the overall failure rate antibiotic treatment in our multivariate analysis, which identified only age >60 and thrombocytopenia < 20000/mm^3 ^as factors. The presence of thrombocytopenia as a risk factor for treatment failure probably reflects the hemotoxicity of chemotherapy. As expected, a univariate analysis revealed that inappropriate antibiotic treatment was a risk factor for first-line treatment failure. However, the relatively low frequency of inappropriate treatment in our study (14% of all patients and 36.4% of the cases of microbiologically documented fever) did not prompt us to change our guidelines on antimicrobial treatment. Our guidelines did not include anti-pseudomonal penicillin or cabapenem contrary as Hughes et al. propose for high risk patients [7]. However our patients were on the border between low and high risk patients. According to the same criteria, our patients had mostly those 4 following criteria for low risk patient: duration of neutropenia less than 7 days, resolution of neutropenia expected in <10 days, no neurological or mental changes, no appearance of illness (40%); in addition, according to the scoring index, most of our patients are in the 20-22 points range. In a very similar population, Rikimaru et al. observed that febrile neutropenia occurred more frequently in patients with poor performance status [17].

The present study had a number of limitations. Firstly, the data were non-exhaustive because of the exclusion of non-hospitalized patients. However, the proportion of cases with febrile neutropenia in our study (13%) is the same as that found by Cullen et al. in a prospective, UK study of patients with solid tumors (cancers and lymphoma). It may be that hospitalization of this type of patient is more frequent in France than in the UK. Secondly, bacteriological samples (especially urine and sputum samples) could have been contaminated and the pathogenic classification of any isolated germs was left to the attending physician in each case. Hence, there may have been confusion bias. Thirdly, this was a retrospective study, with all the weaknesses associated with this type of study design. However, in order to limit selection bias, we selected all consecutive hospitalized lung cancer patients and then excluded those lacking fever and neutropenia. Some patients may have been misclassified. Hence, all medical records charts were carefully reviewed by one of the authors (E.P.) according to pre-established criteria and were classified independently of the initially reported laboratory results.

The febrile neutropenia mortality rate observed here (16%) is greater than the value reported in a similar study (3%). This disparity may be due to the severity of our patients' disease.

As mentioned above, our results revealed a greater proportion of cases with fever of undetermined origin (46%) than those with microbiologically documented fever (38%) and clinically documented fever (16%). However, this difference has been found in other populations. Furthermore, fever of undetermined origin has a better prognosis [14,18,19].

In the present study, microbiologically documented fever was more likely to involve Gram-negative bacteria than Gram-positive bacteria. Furthermore, Gram-negative infections had a worse prognosis for antibiotic treatment than Gram-positive infections did. In contrast to our present results, Cordonnier et al. reported that Gram-positive cocci were predominant. However, a few studies in lung cancer patients have yielded similarly discordant results: Markman and Berghams mainly found Gram-negative bacteria, whereas Matsui and Niho mainly found Gram-positive bacteria [11-13,20]. However, the study populations were somewhat different (Table 5). These disparities do not appear to be time-related (as Oppenheim has suggested for hematologic patients). The high proportion of Gram-negative bacteria could be explained by the absence of aracytin use and antifungal/antimicrobial decontamination (known to facilitate Gram-positive bacterial infections). Furthermore, only three infections were related to a central intravenous catheter (used in 92% of patients), which are known to be primarily due to Gram-positive bacteria. In view of these differences, the results of the above-mentioned studies should be interpreted with a degree of caution and should probably not be generalized to other centers (at least not without knowing more about the microbiological results in each center) but could be extended to lung cancer patients.

**Table 5:** Characteristics of infections in patients with lung cancer: review.

Authors	Population	Patients (n)	Fever (n)	MDF (%)	Gram - (% MDF)	Bacteria	Gram+ (% MDF)	Bacteria
Markman et al.^11^ 1983	Small cell cancer neutropenic	72	126	11	61	*E. coli* *P. aeruginosa*	31	*S. viridans* *S. aureus Bacillus spp*
Matsui et al.^12^ 1991	All lung cancer Leukocytes count <3000/mm3	-	101	58	44	-	56	-
Berghmans et al.^20^ 2003	All lung cancer neutropenic and non neutropenic	275	435	60	64	*H. influenzae* *M. catarrhalis* *E. coli* *P. aeruginosa*	25	*S. pneumoniae* *S. aureus*
Niho et al.^13^ 2004	All lung cancer neutropenic	35	41	12	40	*P. aeruginosa* *H. influenzae*	60	*S. pneumoniae* *S. aureus* *Enterococcus*
Lanoix et al. 2011	All lung cancer neutropenic	87	87	38	58	*E. coli* other Enterobacteriaceae	40	*S. aureus* *S. epidermidis*

MDF: microbiologically documented fever

Klastersky et al. reported on a huge cohort of 2142 patients with febrile neutropenia, of whom 164 had lung cancer [16]. The aim of the latter study was to compare the severity of disease in bacteremic and non-bacteremic patients. The mortality rate was 8% in the small subgroup of lung cancer patients and the data were not analyzed further.

Kern reported that 20% of the infections were multidrug-resistant (versus 14.6% in the present study), with an antibiotic treatment failure rate of 22% (versus 18.2% here) [14]. The mortality rate due to infection was 2.5% (versus 16% here) and the overall mortality rate at day 30 was 5% (versus 33% here). Of course, Kern's study population differed from ours and was considered to be at low risk of infection.

## Conclusion

The present study emphasized two important points. Firstly, the microbiological profile of febrile neutropenia does not differ greatly from that seen in other types of neutropenia. Respiratory germs are less predominant than *S. aureus *and enterobacteriaceae and lung infection is not a prognostic factor (since only age and performance status were predictive of antibiotic treatment failure). Secondly, our results highlight the progression of the infection; the higher mortality rate was due more to the patients' fragility than the nature of the germs or the presence of multidrug resistance. Extremely broad-spectrum antibiotic therapy is not always necessary but treatment must take account of the patient's frailty and the presence of respiratory germs.

In order to circumvent the limitations of retrospective studies and avoid selection bias, it would be useful to perform the same type of study on a prospective basis and include non-hospitalized patients.

## Competing interests

The authors declare that they have no competing interests.

## Authors' contributions

JPL wrote the manuscript, EP carried over the data from medical records, FXL helped to design the study and performed the statistical analysis, HB, EL and MB provided patients from Saint Quentin, PD provided patients from Chauny, VJ provided patients from Amiens, JLS and CD conceived the study and helped to design and manage it, YD conceived the study, helped to design and manage it and helped to draft the manuscript. All authors read and approved the final manuscript.

## Pre-publication history

The pre-publication history for this paper can be accessed here:

http://www.biomedcentral.com/1471-2334/11/183/prepub
